# Supplementary material for: Allele-Specific CG/CCWGG Methylation of the PSA Promoter Discriminates Aggressive, Indolent, and Benign Prostate Cell Lines and Is Involved in the Regulation of PSA Expression
Source: Int J Mol Sci. 2025 Jan 31;26(3):1243. doi: 10.3390/ijms26031243 (PMC11818708; doi:10.3390/ijms26031243)
Supplement: Supplementary file 1 [file ijms-26-01243-s001.zip › ijms-3420778-supplementary.pdf]

Supplementary material

Mikhail Baryshev[1]*, Egils Vjaters[2]

[1]Institute of Microbiology and Virology, Riga Stradins University, Ratsupites Str 5, LV-1067, Riga, Latvia

[2]Institute of Oncology and Molecular Genetics, Riga Stradins University, Pilsoņu Str 13, LV-1007, Riga, Latvia

* Address correspondence to Mihails.Barisevs@rsu.lv

Supplementary figures

Sequencing of DM miniprepped DNA applied for proximal PSA promoter methylation analysis.

Supplementary Fig. S 1. Representative chromatogram of partial CG methylation of the PSA promoter specific for both HPrEpiC alleles.

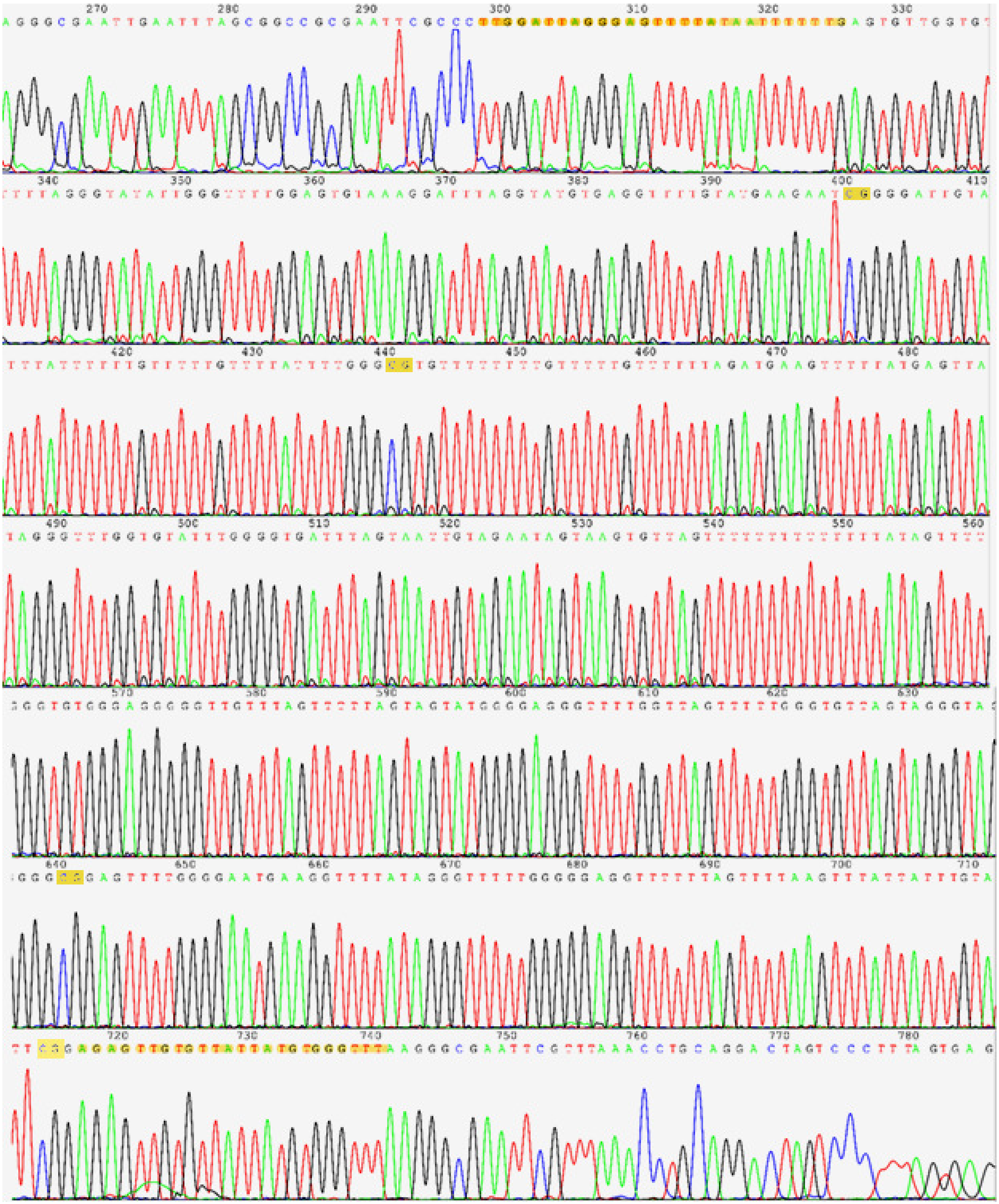

Supplementary Fig. S 2. Representative chromatogram of complete CG methylation of the PSA promoter specific for both PC3 alleles.

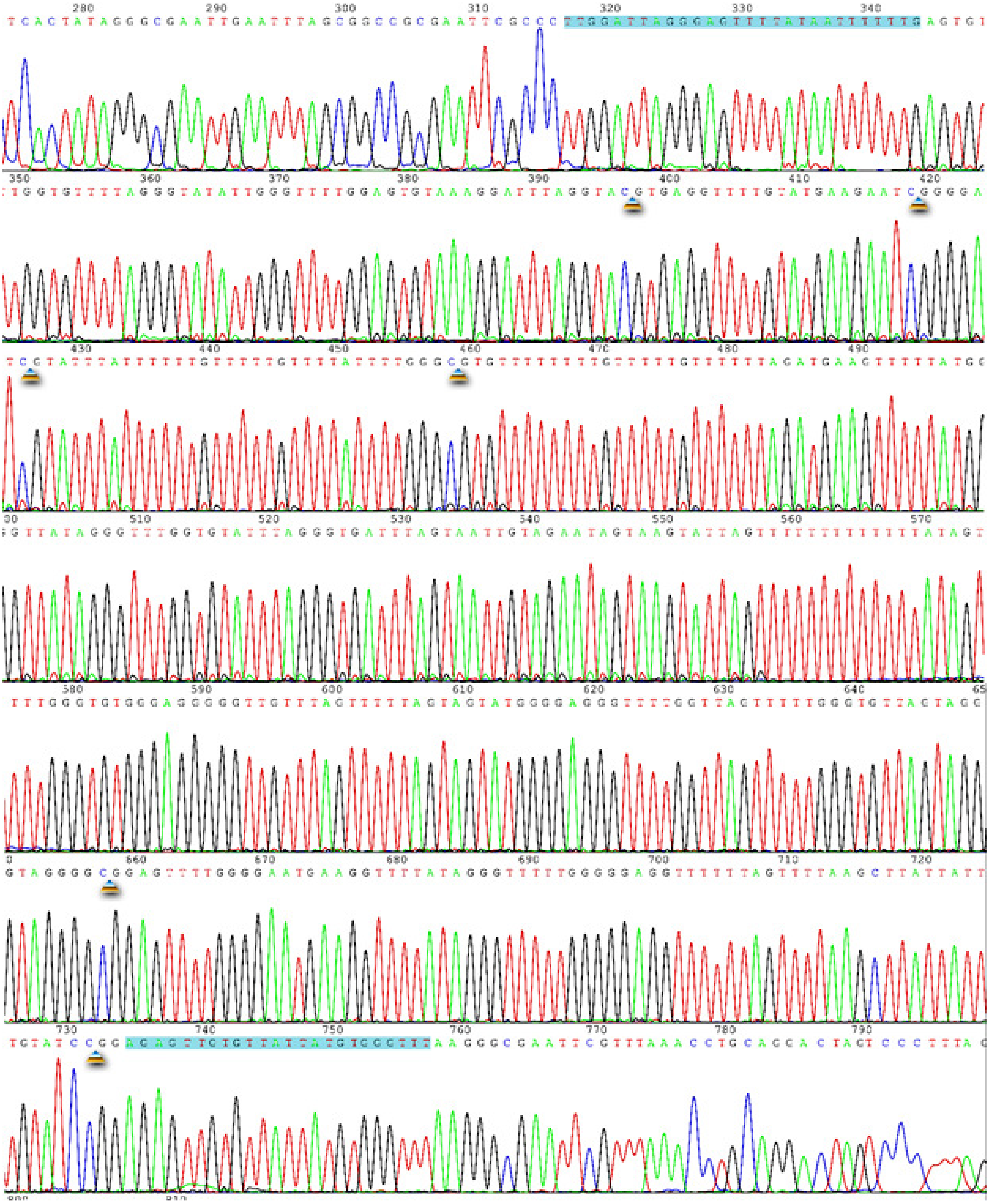

Supplementary Fig. S 3. The RNA expression level in 33 TCGA Cancer Tissues (GENCODE v23) and Genotype-Tissue Expression (GTEx) project data of Gene Expression in 54 tissues from GTEx RNA-seq of 17382 samples, 948 donors (V8, Aug 2019) are presented with UCSC Genome Browser on Human (GRCh38/hg38). Expression for the gene represented by coloured bargraph, where the height of each bar represent the median expression level across all samples for a tissue. The bar colour indicates the tissue.

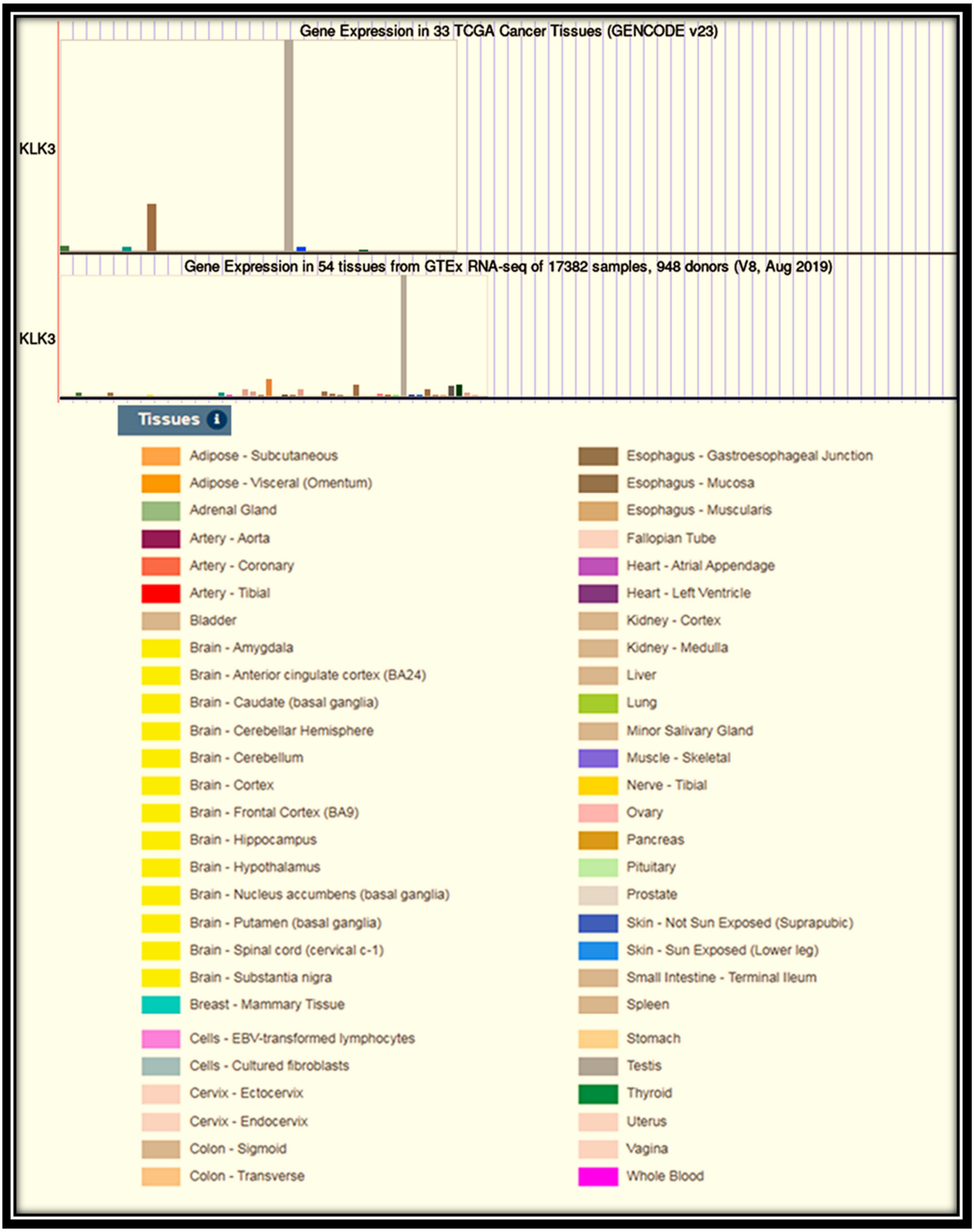
Gene Expression in 33 TCGA Cancer Tissues (GENCODE v23)
KLK3
Gene Expression in 54 tissues from GTEx RNA-seq of 17382 samples, 948 donors (V8, Aug 2019)
KLK3
Tissues
Adipose - Subcutaneous
Adipose - Visceral (Omentum)
Adrenal Gland
Artery - Aorta
Artery - Coronary
Artery - Tibial
Bladder
Brain - Amygdala
Brain - Anterior cingulate cortex (BA24)
Brain - Caudate (basal ganglia)
Brain - Cerebellar Hemisphere
Brain - Cerebellum
Brain - Cortex
Brain - Frontal Cortex (BA9)
Brain - Hippocampus
Brain - Hypothalamus
Brain - Nucleus accumbens (basal ganglia)
Brain - Putamen (basal ganglia)
Brain - Spinal cord (cervical c-1)
Brain - Substantia nigra
Breast - Mammary Tissue
Cells - EBV-transformed lymphocytes
Cells - Cultured fibroblasts
Cervix - Ectocervix
Cervix - Endocervix
Colon - Sigmoid
Colon - Transverse
Esophagus - Gastroesophageal Junction
Esophagus - Mucosa
Esophagus - Muscularis
Fallopian Tube
Heart - Atrial Appendage
Heart - Left Ventricle
Kidney - Cortex
Kidney - Medulla
Liver
Lung
Minor Salivary Gland
Muscle - Skeletal
Nerve - Tibial
Ovary
Pancreas
Pituitary
Prostate
Skin - Not Sun Exposed (Suprapubic)
Skin - Sun Exposed (Lower leg)
Small Intestine - Terminal Ileum
Spleen
Stomach
Testis
Thyroid
Uterus
Vagina
Whole Blood
